# Subcentimeter Pulmonary Nodules Detected in Patients with Sarcoma

**DOI:** 10.1155/S1357714X00000116

**Published:** 2000-03

**Authors:** Michelle S. Ginsberg, David M. Panicek

**Affiliations:** Department of Radiology Memorial Sloan-Kettering Cancer Center 1275 York Avenue NewYork NY 10021 USA

## Abstract

*Background.* Subcentimeter pulmonary nodules are being detected with increasing frequency in patients with sarcoma due to the greater use of chest CT, the advent of helical (spiral) CT scanning and multidetector scanners, and the attendant decrease in image section thickness.Assessing the clinical significance of these pulmonary nodules is of particular importance in sarcoma patients, due to the frequent occurrence of pulmonary metastasis from sarcomas.

*Purpose.* This article reviews the technical advances that have contributed to the increased detection of subcentimeter pulmonary nodules, statistics about subcentimeter pulmonary nodules and options for evaluating such nodules.


*Sarcoma* (2000) **4**, 63–66

ISSUES IN IMAGING

### Subcentimeter pulmonary nodules detected in patients with sarcoma

MICHELLE S. GINSBERG & DAVID M. PANICEK

*Department of Radiology, Memorial Sloan-Kettering Cancer Center, 1275 York Avenue, New York, NY 10021, USA*

**Abstract**

*Background.* Subcentimeter pulmonary nodules are being detected with increasing frequency in patients with sarcoma due to the greater use of chest CT, the advent of helical (spiral) CT scanning and multidetector scanners, and the attendant decrease in image section thickness. Assessing the clinical significance of these pulmonary nodules is of particular importance in sarcoma patients, due to the frequent occurrence of pulmonary metastasis from sarcomas.

*Purpose.* This article reviews the technical advances that have contributed to the increased detection of subcentimeter pulmonary nodules, statistics about subcentimeter pulmonary nodules and options for evaluating such nodules.

**Key words:** *computed tomography (CT), helical CT, pulmonary nodule, pulmonary metastasis, sarcoma*

### Introduction

Subcentimeter pulmonary nodules detected at chest CT in patients with sarcoma can represent a variety of benign processes, including hamartoma, intrapulmonary lymph node, granuloma (calcified or uncalcified), inflammation, fibrosis, tumorlet (a minute benign epithelial lesion encountered as an incidental finding during microscopic examination of the lung), or sterilized metastasis, or they can be due to metastatic sarcoma or even an early primary lung cancer. The relatively high probability of malignancy in pulmonary nodules in patients with a known sarcoma is an important factor in patient management. Recent advances in CT scanning have led to the discovery of even more subcentimeter pulmonary nodules than on conventional CT or chest radiography, often complicating the evaluation of patients with sarcoma.

### Discussion

### *Helical (spiral) CT*

On conventional CT, small pulmonary nodules may not be imaged due to inconsistent depth of patient inspiration on sequential breath holds, or to the relatively thick sections obtained (typically 10 mm). The introduction of helical CT has significantly increased the detection rate of small pulmonary nodules by eliminating respiratory misregistration.[1–3] Helical CT also allows reconstruction of the acquired helical CT data set in overlapping sections, further improving detection of subcentimeter nodules.[3] Moreover, helical CT typically utilizes thinner image sections (e.g. 5–7 mm) than did conventional CT, resulting in sharper images with less partial volume averaging effects.

Although obtaining very thin CT sections (with collimation thinner than 5 mm) might seem to be an attractive option for demonstrating subcentimeter pulmonary nodules, very thin sections can actually make it more difficult to distinguish nodules from adjacent blood vessels. Tiny vascular branches, when sectioned thinly, can mimic tiny pulmonary nodules and, by creating a complex background of tiny nodular opacities, may also prevent identification of any intermingled nodules.

Reconstruction of thin-section helical CT data of the lung can be performed with a sliding thin-slab maximum intensity projection (MIP) technique. This results in a two-dimensional representation of the initial 1-mm thick sections by means of arithmetic calculation of maximum intensities encountered along an arbitrarily oriented mathematic ray. MIP images depict the entire course of a vessel within a selected subvolume, allowing easier identification of a nodule and distinction of it from adjacent vessels.[4,5] This technique is, however, not in routine clinical use at this time.

At many facilities, radiologists are now interpreting CT studies on workstations rather than on radiographic film. Scrolling through helical CT images on a computer workstation in a sequential 'cine' mode

Correspondence to: Michelle S. Ginsberg, MD, Department of Radiology, Memorial Sloan-Kettering Cancer Center, 1275 York Avenue, New York, NY 10021, USA. Tel: +1 (212) 639 7292; Fax: +1 (212) 794 4010; e-mail: ginsberm @ mskcc.org

1357-714X print/1369-1643 online/00/010063-04 © 2000 Taylor & Francis Ltd has distinct technical and perceptual advantages, significantly increasing the detection rate of pulmonary nodules 5 mm in diameter or smaller as compared to film-based viewing; no significant advantage has been demonstrated for lesions larger than 5 mm.[6] On a perceptual level, ciné viewing facilitates differentiation of tubular structures (such as blood vessels) from spherical structures (such as pulmonary nodules) by constructively activating the motion-processing channel in the human visual system.[7]

*Frequency of malignancy in small pulmonary nodules*

Radiologists are often asked to assess the clinical significance of subcentimeter pulmonary nodules, particularly the likelihood that a given nodule is malignant. This assessment is especially important in patients with sarcoma, for whom the lung is the most common site of metastatic disease. Also, resection of pulmonary metastatic disease can be an important factor in determining outcome.[8]

Prior studies have reported probabilities of malignancy in pulmonary nodules based on anecdotal data[9] or autopsy series.[10,11] We recently reviewed the etiology of pulmonary nodules resected at video-assisted thoracoscopic surgery (VATS) in 426 patients. In patients with a known primary malignancy, nodules 5 mm or less were more likely benign (58% of 275 resected pulmonary nodules), whereas pulmonary nodules 5 mm but ≤1 cm were more likely malignant (69% of 149 resected pulmonary nodules; $p<0.001$).[12] Multiple nodules are more likely malignant than a single nodule in patients with a known extrathoracic malignancy.[12,13] In our study, 17 (57%) of 30 small nodules resected from 14 patients known to have a diagnosis of sarcoma were malignant. Nodules ≤1 cm in these patients were equally likely to be benign or malignant: 11 such nodules were benign and 12 were malignant (Figs 1 and 2). Nodules greater than 1 cm, however, were more likely malignant: five such nodules were malignant and two were benign.

*Work-up of pulmonary nodules*

Unless fat or a characteristic pattern of calcification can be demonstrated within a lung nodule, or prior radiological studies demonstrate stability of the nodule for at least 2 years — either of which would indicate the nodule is benign — the nature of the nodule remains obscure at imaging. However, calcification within a lung nodule in patients with sarcoma may be misleading, as metastatic lesions from osteosarcoma or chondrosarcoma can contain radiologically demonstrable calcification (Fig. 3).

Lung nodule enhancement has recently been proposed as an indicator of malignancy and vascularity in small nodules (i.e. those measuring 7–30 mm). Malignant nodules were shown to enhance statistically significantly more than granulomas and benign neoplasms, with a sensitivity of 98%, specificity of 73%, and accuracy of 85%.[14] Blood flow patterns at dynamic CT of malignant, benign and inflammatory solitary pulmonary nodules have

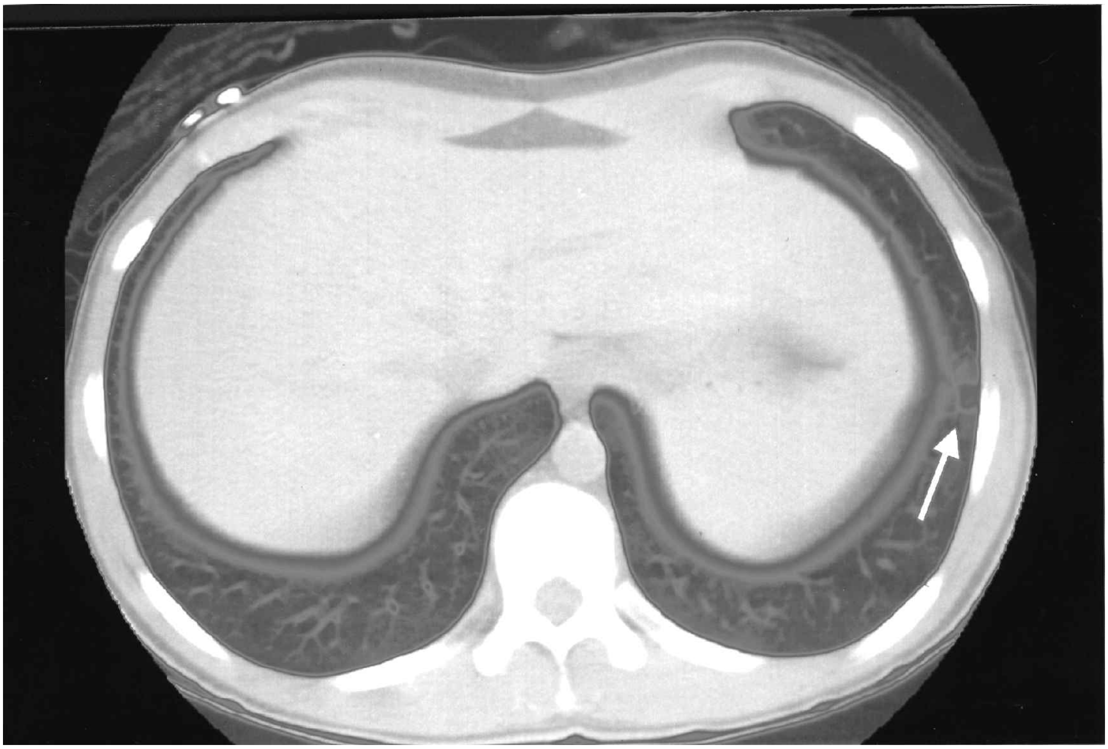

**Figure 1.** *An 18-year-old male with high grade synovial sarcoma of arm diagnosed 3 months earlier. Helical CT shows 0.8×0.6 cm left lower lobe lung nodule (arrow) not visible on prior helical CT. Histopathological examination of wedge resection specimen demonstrated a benign intrapulmonary lymph node.*

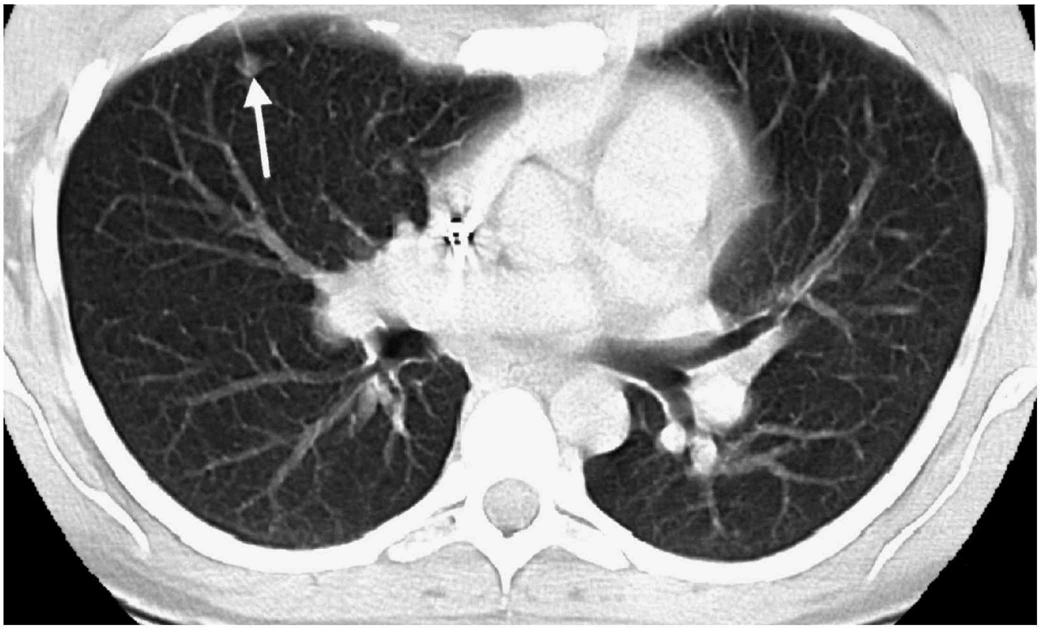

**Figure 2**. *A 21-year-old female with Ewing's sarcoma of left pelvis. CT at presentation showed three subcentimeter lung nodules. Helical CT image demonstrates one 0.7×0.6 cm nodule (arrow) in the right middle lobe, which at resection represented a benign lymph node. All three nodules were benign at biopsy, demonstrating alveolar fibrosis, atelectasis and unremarkable lymphoid tissue.*

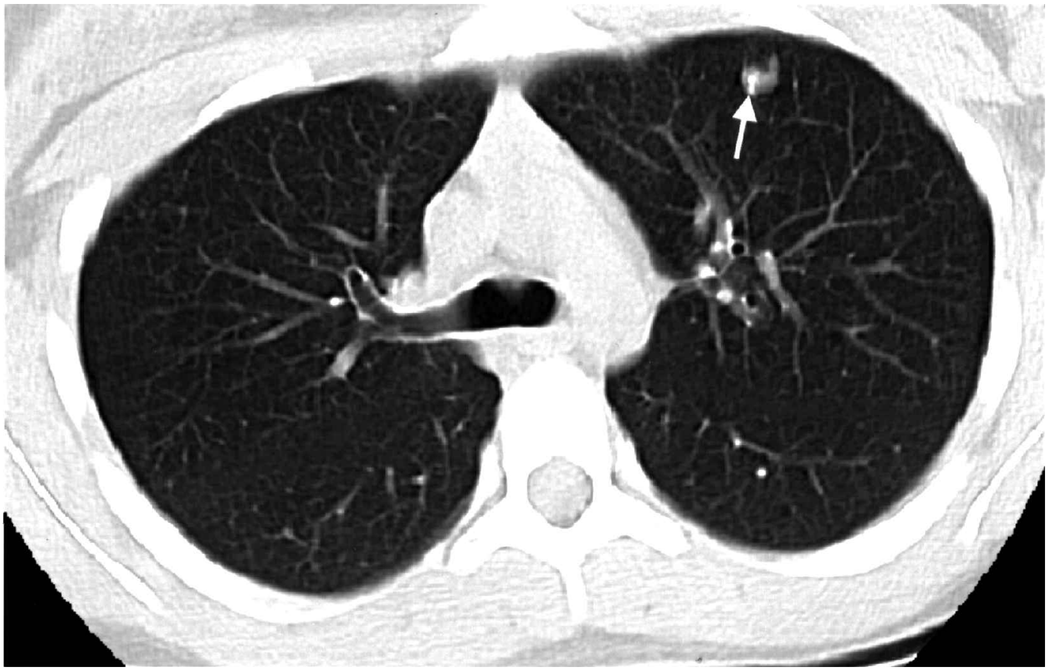

**Figure 3**. *A 16-year-old female with osteosarcoma of distal femur diagnosed 3 years earlier. Helical CT shows 1×0.8 cm partly calcified pulmonary nodule (arrow) in left upper lobe. Histopathological examination of wedge resection specimen showed osteogenic sarcoma (50% viable tumor).*

recently also been evaluated. Although there was a significant difference among overall groups of malignant and inflammatory nodules as compared to benign nodules, no statistically significant difference was found between malignant and inflammatory nodules.[15] Also, there was some overlap in findings of individual benign and malignant nodules, limiting the usefulness of this technique for evaluation of an individual patient.

Assessment for nodule growth with early repeat CT has recently been studied. Early repeat CT reportedly can depict a size change within 30 days for malignancies with doubling times of 30–180 days and that are larger than 5 mm at initial presentation; many of these nodule measurements, however, were made on phantom models.[16] This technique is not available for routine clinical use at this time, but likely will soon become widespread. Unless follow-up CT at longer time intervals is a clinically viable option in a given patient, a subcentimeter pulmonary nodule detected at CT may need to be biopsied, either percutaneously, at VATS, or at open biopsy.

Positron emission tomography (PET) is a powerful radiological technique that can distinguish benign and malignant nodules in many cases. However, PET currently has limited ability to assess subcentimeter lesions.

### Conclusion

The detection of more subcentimeter pulmonary nodules at CT is a mixed blessing in patients with sarcoma. Although some tiny pulmonary metastases will be discovered earlier, there currently is no routine non-invasive imaging method that allows accurate distinction between benign and malignant pulmonary nodules of that size. Subcentimeter pulmonary nodules are more likely benign, even in patients with a known malignancy. Close follow-up with sequential chest CT scans to assess for interval growth is one viable option, especially in those sarcomas that exhibit rapid growth; alternatively, biopsy may be required for definitive evaluation.

### References

1 Kalender WA, Polacin A, Suss C. A comparison of conventional and spiral CT: an experimental study on the detection of spherical lesions. *J Comp Assiot Tomog* 1994;18:167–76.

2 Remy-Jardin M, Remy J, Giraud F, Marquette CH. Pulmonary nodules: detection with thick spiral CT versus conventional CT. *Radiology* 1993;187:513–20.

3 Urban BA, Fishman EK, Kuhlman JE, Kawashima A, Hennessey JG, Siegelman SS. Detection of focal hepatic lesions with spiral CT: comparison of 4- and 8-mm interscan spacing. *AJR* 1993;160:783–85.

4 Bhalla M, Nadich DP, McGuinness G, *et al.* Diffuse lung disease: assessment with helical CT-preliminary observations of the role of maximum and minimum intensity projection images. *Radiology* 1996;200:341–47.

5 Napel S, Rubin GD, Jeffrey RB. STS-MIP: a new reconstruction technique for CT of the chest (technical note). *J Comput Assist Tomogr* 1993;17:832–8.

6 Tillich M, Kammerhuber F, Reittner P, Riepl T, Stoeffler G, Szolar D. Detection of pulmonary nodules with helical CT: comparison of cine and film-based viewing. *AJR* 1997;169:1611–14.

7 Seltzer SE, Judy PFJ, Adams DF, *et al.* Spiral CT of the chest: comparison of cine and film-based viewing. *Radiology* 1995;197:73–8.

8 Billingsley KG, Burt ME, Jara E, *et al.* Pulmonary metastases from soft tissue sarcoma. *Ann Surg* 1999;229:602–12.

9 Cahan WG, Castro EB, Hajdu SI. The significance of a solitary lung shadow in patients with colon carcinoma. *Cancer* 1974;33:414–421.

10 Abrams HL, Spiro R, Goldstein N. Metastases in carcinoma. Analysis of 1000 autopsied cases. *Cancer* 1950;3:74–85.

11 Crow J, Slavin G, Kreel L. Pulmonary metastasis: a pathologic and radiologic study. *Cancer* 1982;47:2595–602.

12 Ginsberg MS, Griff SK, Go BD, Yoo HH, Schwartz LH, Panicek DM. Pulmonary nodules resected at video-assisted thoracoscopic surgery: etiology in 426 patients. *Radiology* 1999;213:277–82.

13 Libshitz HI, Peuchot M. Pulmonary metastatic disease: radiologic–surgical correlation. *Radiology* 1987; 164:719–22.

14 Swensen SJ, Brown LR, Colby TV, Weaver AL, Midthun DE. Lung nodule enhancement at CT: prospective findings. *Radiology* 1996;201:447–55.

15 Zhang M, Kono M. Solitary pulmonary nodules: evaluation of blood flow patterns with dynamic CT. *Radiology* 1997;205:471–8.

16 Yankelevitz DF, Gupta R, Zhao B, Henschke CI. Small pulmonary nodules: evaluation with repeat CT-preliminary experience. *Radiology* 1999;212:561–6.
